# Biting Midges (Diptera: Ceratopogonidae) from Cambay Amber Indicate that the Eocene Fauna of the Indian Subcontinent Was Not Isolated

**DOI:** 10.1371/journal.pone.0169144

**Published:** 2017-01-11

**Authors:** Frauke Stebner, Ryszard Szadziewski, Hukam Singh, Simon Gunkel, Jes Rust

**Affiliations:** 1 Steinmann-Institut, Abteilung Paläontologie, Bonn, Germany; 2 University of Gdańsk, Department of Invertebrate Zoology and Parasitology, Gdańsk, Poland; 3 Birbal Sahni Institute of Palaeosciences, Lucknow, India; Royal Belgian Institute of Natural Sciences, BELGIUM

## Abstract

India’s unique and highly diverse biota combined with its unique geodynamical history has generated significant interest in the patterns and processes that have shaped the current distribution of India’s flora and fauna and their biogeographical relationships. Fifty four million year old Cambay amber from northwestern India provides the opportunity to address questions relating to endemism and biogeographic history by studying fossil insects. Within the present study seven extant and three fossil genera of biting midges are recorded from Cambay amber and five new species are described: *Eohelea indica* Stebner & Szadziewski n. sp., *Gedanohelea gerdesorum* Stebner & Szadziewski n. sp., *Meunierohelea cambayana* Stebner & Szadziewski n. sp., *Meunierohelea borkenti* Stebner & Szadziewski n. sp., and *Meunierohelea orientalis* Stebner & Szadziewski n. sp. Fossils of species in the genera *Leptoconops* Skuse, 1889, *Forcipomyia* Meigen, 1818, *Brachypogon* Kieffer, 1899, *Stilobezzia* Kieffer, 1911, *Serromyia* Meigen, 1818, and *Mantohelea* Szadziewski, 1988 are recorded without formal description. Furthermore, one fossil belonging to the genus *Camptopterohelea* Wirth & Hubert, 1960 is included in the present study. Our study reveals faunal links among Ceratopogonidae from Cambay amber and contemporaneous amber from Fushun, China, Eocene Baltic amber from Europe, as well as the modern Australasian and the Oriental regions. These findings imply that faunal exchange between Europe, Asia and India took place before the formation of Cambay amber in the early Eocene.

## 1. Introduction

Modern India is characterized by a diverse biota with many endemic elements especially in the area of the Western Ghats, one of only two terrestrial biodiversity “hotspots” in South Asia [1]. To explain India’s diversity, various biogeographic models have been developed.

The “Biotic ferry” model postulates that India formed an isolated continent for at least 30 Ma after its separation from Madagascar and before its collision with Asia [2, 3], allowing a highly diverse and endemic biota to develop. Common Asian and Indian faunal elements are explained by the “Out of India” and “Out of Asia” theories respectively. In contrast to the “Biotic ferry” theory, models have been developed in which broad land bridge connections between drifting India and Africa or island arcs between India and Asia or India and Africa existed, allowing faunal exchange [3, 4, 5]. The plausibility of these different models strongly depends on geodynamic reconstructions and the precise timing of the India-Asia collision. Age estimates of the initial contact between India and the remainder of Asia range from 70–65 Ma to as recently as 25–20 Ma (e.g. [4, 6, 7, 8, 9, 10, 11]).

Cambay amber derives from active lignite mines in the state of Gujarat, India, about 30 km northeast of Surat. The amber bearing sediments have been dated to early Eocene age (52–55 Ma) [12, 13, 14, 15] and more precisely, terrestrial vertebrate remains from between the two major amber layers in Vastan mine have been estimated at 54.5 Ma [16]. Thus, formation of the amber is within the collision estimations of the Indian subcontinent with the remainder of Asia. Cambay amber fossils are of particular importance since they provide a window to India’s diversity at that time and can facilitate understanding of India’s geological history. Initial studies of inclusions have revealed little faunal isolation of the Indian subcontinent [17]. Rather, other insects in Cambay amber have shown connections to Eocene European Baltic amber (48–43 Ma; [18, 19, 20]) and to the Miocene as well as the modern Neotropics [21, 22]. Interestingly, so far only the whipspiders (Amblypygi) and webspinners (Embioptera) from Cambay amber show biogeographic affinities to Africa and to Africa and South America respectively, indicating a possible Gondwanan origin for these taxa [23, 24].

Ceratopogonidae is one of the most abundant dipteran groups in Cambay amber and one of the most diverse families of Diptera both in the fossil record as well as in modern ecosystems. Currently, there are 283 fossil and 6267 extant species recorded from all over the world [25]. The fossil record of Ceratopogonidae dates back to the Early Cretaceous with findings in the Purbeck limestone of Great Britain (142 Ma) [26], in amber from Jordan and Lebanon (125–129 Ma) [27, 28], in the Koonwarra fossil bed of Australia (113–116 Ma) [29], and in amber from Spain (110–113 Ma) [30] (see also [25, 28]). Late Cretaceous records are from amber from Myanmar (Burmese amber, 98–99 Ma), France (Vendean amber: 85–97 Ma; Charantese amber: late Albian—early Cenomanian), Taimyr (Yantardakh: Santonian; Agapa River: late Cenomanian), New Jersey (90–94 Ma), Hungary (Campanian-Santonian), and Canada (76–80 Ma) (summarized in [25, 28). The earliest Paleogene amber inclusions are early Eocene fossils from India (54 Ma) and Fushun (50–53 Ma) followed by middle Eocene amber of Sakhalin (e.g. [31]). With 26 genera and 109 species recorded [32, 33] the middle to late Eocene Baltic amber fauna is the best studied fossil assemblage. The youngest amber fossils are from the Miocene of Mexico (15–20 Ma) [34, 35] and the Dominican Republic (15–20 Ma) [36].

In the present work Ceratopogonidae from Cambay amber were systematically studied, compared to contemporaneous amber faunas from Fushun and the Baltic Region, and analyzed with respect to their palaeobiogeography. Since Cambay amber was formed at a climatically pivotal period, at the end of the Paleocene Eocene Thermal Maximum and the beginning of the Early Eocene Climatic Optimum (e.g. [37]), the fossils were also examined relative to their palaeoecological implications.

## 2. Materials and Methods

The study is based on 38 specimens of Ceratopogonidae in early Eocene Cambay amber from India (Table 1). Syninclusions are listed separately in S1 Table. Photos of Ceratopogonidae specimens from Cambay amber without formal descriptions can be found in S1 and S2 Figs. Eight fossil Ceratopogonidae from contemporaneous Fushun amber, deposited at the Nanjing Institute of Geology and Palaeontology, Chinese Academy of Sciences, Nanjing, China are included in the biogeographic discussion. Cambay amber specimens derive from lignite mines in Tadkeshwar (N 21° 21.400, E 073° 04.532), Vastan (N 21° 25.239, E 073° 07.249) and Valia (21°24.690, 073°05.939) in Gujarat, India. Samples are from the collections of the Steinmann Institute, Bonn, Germany and the American Museum of Natural History (AMNH), New York, USA. Both collections have been amassed without any collector’s bias so that Ceratopogonidae composition recorded reflects the actual faunal elements entrapped in the collected amber material. All specimens will be deposited in the collection of the AMNH. All necessary permits were obtained for the described study, which complied with all relevant regulations.

**Table 1:** Ceratopogonidae in Cambay amber, deposited in the collection of the American Museum of Natural History. Abbreviations: H = holotype, P = paratype.

Inventory no.	Deposit	Subfamily	Genus	Species	sex
Tad-852 a	Tadkeshwar	Leptoconopinae	*Leptoconops*	indet.	♂
Tad-506	Tadkeshwar	Leptoconopinae	*Leptoconops*	indet.	♂
Tad-163	Tadkeshwar	Forcipomyiinae	*Forcipomyia*	indet.	♀
Tad-511 a	Tadkeshwar	Forcipomyiinae	*Forcipomyia*	indet.	♀
Tad-565	Tadkeshwar	Forcipomyiinae	*Forcipomyia*	indet.	♀
Tad-602	Tadkeshwar	Forcipomyiinae	*Forcipomyia*	indet.	♀
Tad-616 a	Tadkeshwar	Forcipomyiinae	*Forcipomyia*	indet.	♀
Tad-643	Tadkeshwar	Forcipomyiinae	*Forcipomyia*	indet.	♀
Tad-862 a	Tadkeshwar	Forcipomyiinae	*Forcipomyia*	indet.	♀
Val-3.3	Valia	Forcipomyiinae	*Forcipomyia*	indet.	♀
Vas-48	Vastan	Forcipomyiinae	*Forcipomyia*	indet.	♀
Tad-511 b	Tadkeshwar	Forcipomyiinae	*Forcipomyia*	indet.	♂
Tad-856	Tadkeshwar	Forcipomyiinae	*Forcipomyia*	indet.	♂
Tad-860	Tadkeshwar	Forcipomyiinae	*Forcipomyia*	indet.	♂
Val-3.4	Valia	Forcipomyiinae	*Forcipomyia*	indet.	♂
Val-3.6	Valia	Forcipomyiinae	*Forcipomyia*	indet.	♂
Tad-508	Tadkeshwar	Ceratopogoninae	*Brachypogon*	indet.	♀
Tad-854	Tadkeshwar	Ceratopogoninae	*Brachypogon*	indet.	♀
Tad-851	Tadkeshwar	Ceratopogoninae	*Brachypogon*	indet.	♂
Val-3.5	Valia	Ceratopogoninae	*Meunierohelea*	indet.	♀
Tad-507	Tadkeshwar	Ceratopogoninae	*Meunierohelea*	*Meunierohelea cambayana* n. sp. (H)	♂
Tad-516	Tadkeshwar	Ceratopogoninae	*Meunierohelea*	*Meunierohelea borkenti* n. sp. (H)	♂
Tad-519	Tadkeshwar	Ceratopogoninae	*Meunierohelea*	*Meunierohelea* af *cambayana*	♂
Tad-858 a	Tadkeshwar	Ceratopogoninae	*Meunierohelea*	*Meunierohelea orientalis*n. sp. (H)	♂
Tad-673	Tadkeshwar	Ceratopogoninae	*Mantohelea*	indet.	♀
Tad-513	Tadkeshwar	Ceratopogoninae	*Gedanohelea*	*Gedanohelea gerdesorum* n. sp. (P)	♀
Tad-661	Tadkeshwar	Ceratopogoninae	*Gedanohelea*	*Gedanohelea gerdesorum* n. sp. (P)	♀
Tad-857	Tadkeshwar	Ceratopogoninae	*Gedanohelea*	*Gedanohelea gerdesorum* n. sp. (H)	♀
Val-3.2	Valia	Ceratopogoninae	*Gedanohelea*	*Gedanohelea gerdesorum* n. sp. (P)	♀
Tad-853 a	Tadkeshwar	Ceratopogoninae	*Stilobezzia*	indet.	♀
Tad-515 a	Tadkeshwar	Ceratopogoninae	*Stilobezzia*	indet.	♂
Tad-518	Tadkeshwar	Ceratopogoninae	*Stilobezzia*	indet.	♂
Tad-647	Tadkeshwar	Ceratopogoninae	*Serromyia*	indet.	♂
Tad-855	Tadkeshwar	Ceratopogoninae	*Eohelea*	*Eohelea indica* n. sp. (H)	♀
Tad-859 a	Tadkeshwar	Ceratopogoninae	*Camptopterohelea*	*Camptopterohelea odora* Stebner et al., 2016 (H)	♀
Tad-615	Tadkeshwar	indet.			♀
Tad-684	Tadkeshwar	indet.			♀
Vas-106	Vastan	indet.			♀

Amber pieces were ground using a Buehler Phoenix Beta grinding machine. For taxonomic identification and investigation a Leica MZ 12_5_ stereoscope was used. Photographs were taken with an “AXIO Zoom.V16 Stereomicroscope” (Carl Zeiss, Jena) equipped with an “AXIOCam HRc Digital Camera” (Zeiss), using the “extended depth of focus” function and with the classic microscope PZO Biolar SK14 and the Helicon Focus 6 image stacking software.

Terms for morphological structures follows those used in the Manual of Nearctic Diptera [38]; special morphological terms and abbreviations follow those explained by Szadziewski [32, 39]. The female antennal ratio (AR) is obtained by dividing the combined length of the distal five flagellomeres by the combined length of the proximal eight flagellomeres; that of the male is obtained by dividing the combined length of the distal three flagellomeres by the combined length of the proximal 10 flagellomeres; the tarsal ratio of the fore leg TR (I), mid leg TR (II) and hind leg TR (III) is obtained by dividing the length of the respective first tarsomere by the length of the second tarsomere; the costal ratio (CR) is calculated by dividing the length of the costa by wing length as measured from the arculus. Wing cells and veins are abbreviated as follows: br = basal radial cell; C = costal vein; CuA_1_, CuA_2_ = branches of cubital vein; M_1_, M_2_ = branches of medial vein; R_1_, R_3_ = branches of radial vein; r_1_ –r_3_ = radial cells; r-m = radial-medial crossvein; Sc = subcostal vein.

Drawings were made using Adobe Illustrator CS6; photo-plates were edited using Photoshop CS5.1 and Adobe Illustrator CS6. Data in the figure showing relationships of select extant and fossil Ceratopogonidae genera was plotted using R [40] and the phytools package [41].

There still is controversy about the age and origin of Baltic, Bitterfeld (Saxonian) and Rovno amber and whether they are contemporaneous. Since most of the aforementioned ambers have been treated and labeled as Baltic amber, which makes an exact assignment impossible, the term Baltic amber is used in the present study for amber deriving from all three localities.

Nomenclatural Acts

The electronic edition of this article conforms to the requirements of the amended International Code of Zoological Nomenclature, and hence the new names contained herein are available under that Code from the electronic edition of this article. This published work and the nomenclatural acts it contains have been registered in ZooBank, the online registration system for the ICZN. The ZooBank LSIDs (Life Science Identifiers) can be resolved and the associated information viewed through any standard web browser by appending the LSID to the prefix “http://zoobank.org/”. The LSID for this publication is: urn:lsid:zoobank.org:pub:F92B27AC-F83B-41B4-802E-A60EEF245643. The electronic edition of this work was published in a journal with an ISSN, and has been archived and is available from the following digital repositories: PubMed Central, LOCKSS.

## 3. Results

Within the present study 34 of the 38 fossil biting midges from Cambay amber could be assigned to one of three subfamilies: Leptoconopinae (2 specimens), Forcipomyiinae (14 specimens), Ceratopogoninae (18 specimens) (Table 1). All of these fossils are determined to one of six extant and three fossil genera: *Forcipomyia* Meigen (14 specimens), *Meunierohelea* Szadziewski (5), *Brachypogon* Kieffer (3), *Stilobezzia* Kieffer (3), *Leptoconops* Skuse (2), *Serromyia* Meigen (1), †*Mantohelea* Szadziewski (1), †*Eohelea* Szadziewski (1) and †*Gedanohelea* Szadziewski (4). One fossil belonging to the genus *Camptopterohelea* Wirth & Hubert has been described in an earlier work [42].

In addition to three previously described species from the genera *Gedanohelea* and *Mantohelea* [43] the study of five newly studied fossils from early Eocene Fushun amber yielded three fossils of the genus *Atrichopogon* Kieffer and two undetermined Forcipomyiinae.

### Systematic palaeontology

**Family** Ceratopogonidae Newman, 1834

**Subfamily** Ceratopogoninae

**Tribe** Ceratopogonini

**Genus** *Eohelea* Petrunkevitch, 1957

#### Type species

*Eohelea stridulans* Petrunkevitch, 1957

#### Diagnosis

Male with 12 flagellomeres, terminal 4 flagellomeres elongate, plume not developed. Wing in both sexes with cell r_1_ short, cell r_2_elongate, costa prolonged nearly to wing apex. Female wing either with elliptic to circular wing patch (commonly: “stridulating organ”) in anterodistal portion of cell r_3_ and vein M_1_ absent distally or without wing patch and vein M_1_ complete; claws short, equal, each with basal inner tooth.

#### Distribution

Six fossil species in Eocene amber from the Baltic Region (*E*. *sinuosa* (Meunier 1904); *E*. *petrunkevitchi* Szadziewski, 1984; *E*. *grogani* Szadziewski, 1988; *E*. *gedanica* Szadziewski, 1988; *E*. *fossicola* Szadziewski, 1993; *E*. *miocaenea* Szadziewski, 1993) and one fossil species in Eocene Sakhalin amber (*E*. *sakhalinica* Szadziewski, 1990).

*Eohelea indica* Stebner & Szadziewski n. sp.

urn:lsid:zoobank.org:act:3DC2CD4E-0D68-4D72-A201-1BF7A2300F51

**Fig 1:**
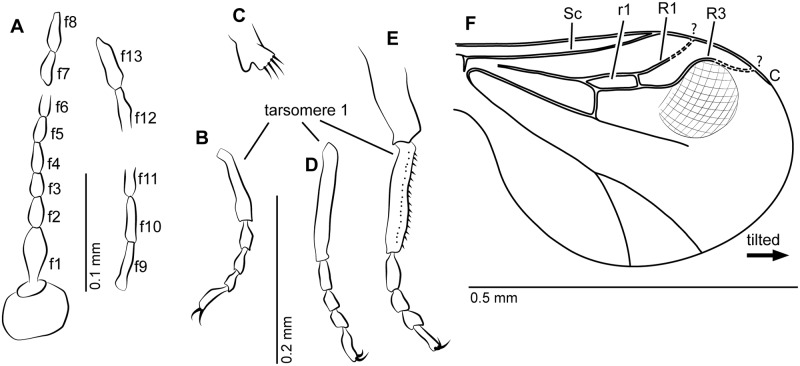
Female of *Eohelea indica* n. sp. from early Eocene Cambay amber, holotype female, no. Tad-855. **A**. Antenna, pedicel and flagellomeres 1–13 (f1-f13). **B**. Tarsus of fore leg. **C**. Tibial comb of hind leg. **D**. Tarsus of mid leg. **E**. Tibia and tarsus of hind leg. **F**. Wing.

**Fig 2:**
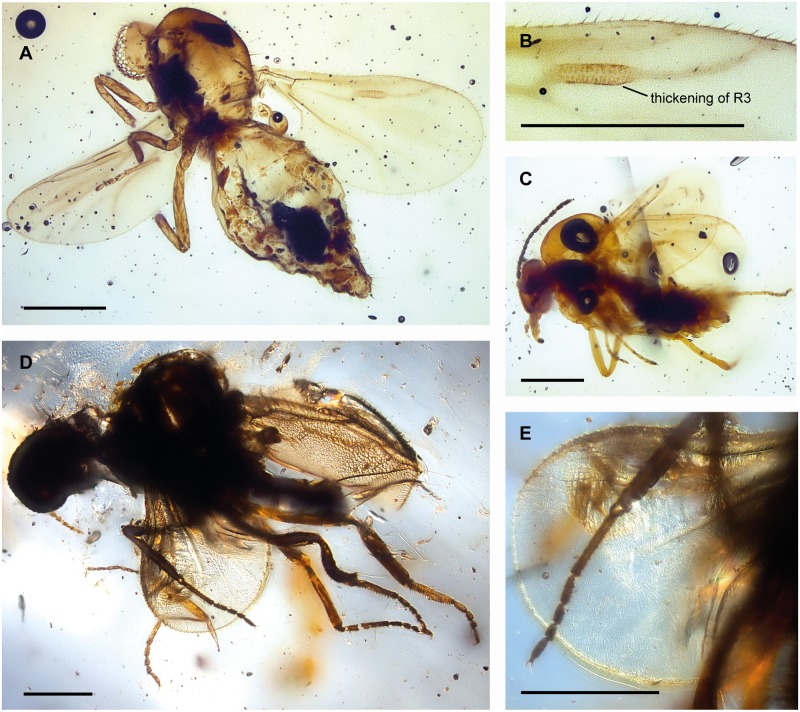
Females of *Gedanohelea gerdesorum* n. sp. and *Eohelea indica* n. sp. from early Eocene Cambay amber. **A**. Habitus of *G*. *gerdesorum*, holotype no. Tad-857. **B**. Radial sector of *G*. *gerdesorum* with thickening of radial vein R_3_, holotype no. Tad-857. **C**. Habitus of *G*. *gerdesorum*, paratype no. Tad-513. **D**. Habitus of *Eohelea indica*, holotype no. Tad-855. **E**. Left wing and mid leg of *Eohelea indica*, holotype no. Tad-855. Scale bars: 0.2 mm.

Figs 1A–1F and 2D and 2E

#### Type material

Holotype female: No. Tad-855, in amber from India: Gujarat, Tadkeshwar lignite mine, Cambay Formation (lower Eocene, Ypresian); in a box labelled “*Eohelea indica* Stebner & Szadziewski. **HOLOTYPE** AMNH IN-Tad-855. Cambay Form. Gujarat India”.

#### Diagnosis

Female: only species of *Eohelea* with a very long subcosta extending beyond apex of cell r_1_ and a circular wing patch that does not extend to the wing apex.

#### Description

Female. Poorly preserved. Body length 1 mm.

*Head*. Eyes separated. Right antenna not preserved, left antenna broken beyond flagellomere 6 (Fig 1A); AR about 1.0 (approximated since antenna is broken and flagellomere 6 is not completely preserved); flagellomeres 2–8 subcylindrical; sensilla coeloconica absent. Proboscis short. Palpus short, with 5 segments.

*Wing* (Fig 1F). Broad, tilted, therefore length approximately 0.48 mm, CR 0.9, vein C nearly reaching wing apex. Vein Sc very long, reaching wing margin beyond level of cell r_1_. Cell r_1_ short, veins R_1_ and R_3_ not visible/preserved distally, thus cell r_2_ not preserved. Wing patch in shape of nearly circular field of wing membrane just below vein R_3_ (only visible in left wing) appears cross-hatched. Vein M_1_ absent distally, vein M_2_ absent. Veins CuA_1_, CuA_2_ well developed, wing membrane without macrotrichia.

*Legs* (Fig 1B–1E). Slender; each with two short equal claws, each armed with basal ventral tooth; fourth tarsomeres subcylindrical; TR(I) 2.5, TR(II) 3.8, TR(III) 2.9. Tibial comb of hind leg with at least 4 spines (Fig 1C), tarsomere 1 of hind leg with two rows of palisade setae (Fig 1E).

*Genitalia*. Cercus short.

Male unknown.

#### Etymology

Specific name refers to the origin of Cambay amber from India.

#### Discussion

*Eohelea indica* n. sp. can be distinguished from all other species of this fossil genus by the very long subcosta. The female of the new species resembles that of *E*. *sinuosa*, *E*. *petrunkevitchi*, *E*. *fossicola*, *E*. *miocaenea* and *E*. *sakhalinica* in having a circular or elliptic, variously structured wing patch just below vein R_3_. The wing patch of *E*. *indica* most closely resembles that of *E*. *fossicola* from Baltic amber. The latter species has a circular patch covered with “finely wrinkled wing membrane” [44] whereas *E*. *indica* has a cross-hatched patterning. All of the wing membrane of both wings of *E*. *indica* n. sp. has an irregular patterning that is most probably a taphonomic result (perhaps caused by infiltration from a subsequent flow of resin after initial capture) and therefore the patterning of the wing patch might also be an artefact and therefore not be useful in distinguishing the two species. Nevertheless, the wing patch of *E*. *indica* is clearly smaller and does not extend close to the wing apex as it does in *E*. *fossicola*.

**Genus** *Gedanohelea* Szadziewski, 1988

#### Type species

*Gedanohelea loewi* Szadziewski, 1988

#### Diagnosis

Wing broad with distinct anal lobe; only first radial cell present; media petiolate, strongly divergent; M_1_ distinctly bowed upward; female claws single and long.

#### Distribution

This fossil genus is known from three species in Eocene Baltic amber (*G*. *wirthi* Szadziewski, 1988; *G*. *loewi* Szadziewski, 1988; *G*. *succinea* Szadziewski, 1988) and two species in early Eocene Fushun amber (*G*. *fushunensis* Stebner et al., 2016; *G*. *liaoningensis* Stebner et al., 2016).

*Gedanohelea gerdesorum* Stebner & Szadziewski n. sp.

urn:lsid:zoobank.org:act:09FD7F99-40C5-4BB2-9984-5D081E6C98BE

**Fig 3:**
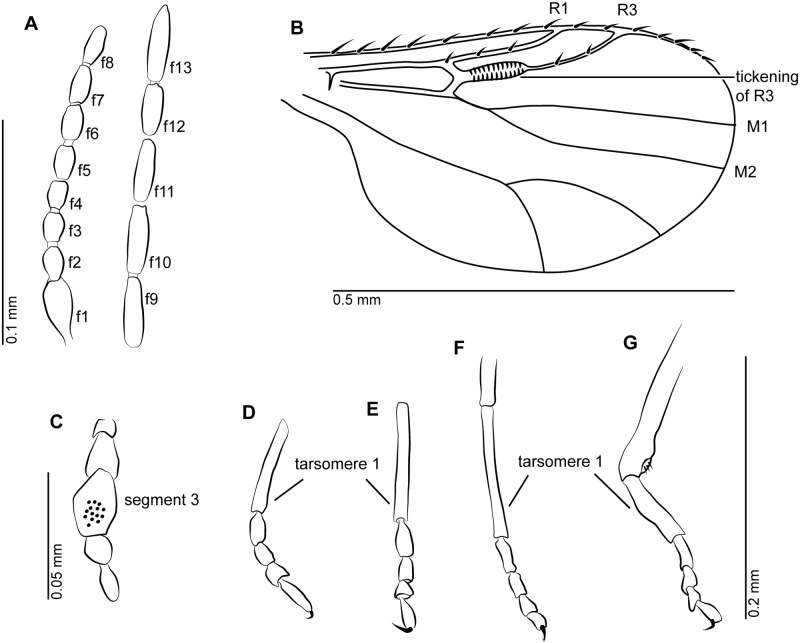
Female of *Gedanohelea gerdesorum* n. sp. from early Eocene Cambay amber. **A**. Flagellomeres 1–13 (f1-f13), paratype no. Tad-513. **B**. Wing, holotype no. Tad-857 **C**. Palpus, paratype no. Tad-513. **D**. Tarsus of fore leg, paratype no. Tad-513. **E**. Tarsus of mid leg, holotype no. Tad-857. **F**. Tarsus of mid leg, paratype no. Tad-513. **G**. Tibia and tarsus of hind leg, paratype no. Tad-513.

Figs 2A–2C and 3A–3G,

#### Type material

Holotype female: No. Tad-857, in amber from India: Gujarat, Tadkeshwar lignite mine, Cambay Formation (lower Eocene, Ypresian); in a box labelled “*Gedanohelea gerdesorum* Stebner & Szadziewski. **HOLOTYPE** AMNH IN-Tad-857. Cambay Form. Gujarat India”. Paratypes, 3 females: Tad-513, Tad-661, Val-3 a.

#### Diagnosis

Female: only species of *Gedanohelea* with wing vein R_3_ basally with peculiar thickening that has several vertical running slit-like lines, and with the first radial cell single, long and broad.

#### Description

Female. Body length 0.8–0.9 mm.

*Head*. Eyes separated. Both antennae missing in holotype, paratypes with 13 flagellomeres (Fig 3A), length of flagellum 0.35 mm, AR 1.06; flagellomeres 2–8 gradually increasing in length; sensilla coeloconica absent. Proboscis short. Palpus missing in holotype, in paratype Tad-513 palpus very short, with 5 segments, segment 3 with sensory pit (Fig 3C).

*Thorax*. Scutellum with 4 marginal setae.

*Wing* (Fig 3B). Broad, length 0.50 mm; CR 0.9; vein R_3_ basally bearing peculiar thickening with several vertical running thin lines. Cell r_1_ long, single; veins M_1_, M_2_ well visible, petiolate; veins CuA_1_, CuA_2_ well developed. Wing membrane covered with distinct microtrichia, macrotrichia absent.

*Legs* (Fig 3D–3G). Broken, partly missing in holotype; in paratypes slender, each armed with single long claw, fourth tarsomeres cordiform, TR(I) 3.2, TR(II) 3.5, TR(III) 3.6.

*Abdomen*. With short cercus.

Male unknown.

#### Etymology

The species name is dedicated to the women in the first author’s family: her mother Heike Gerdes, aunt Karin Gerdes and grandmother Christine Gerdes.

#### Discussion

Because the view of the mid leg of the holotype Tad-857 is distorted the first tarsomere appears shortened when compared to that of the paratype Tad-513.

Females of *Gedanohelea gerdesorum* n. sp. can be easily distinguished from other species of the genus by the unique thickening of the vein R_3_, which is unmodified in all other members of the genus and represents a unique character in the whole family Ceratopogonidae.

**Genus** *Meunierohelea* Szadziewski, 1988

#### Type species

*Meunierohelea nielseni* Szadziewski, 1988

#### Diagnosis

Wing with cells r_1_, r_2_ widely separated by anastomosed R_1_ and R_3_ veins, rarely cell r_1_ absent, base of M_2_ absent.

#### Distribution

One extant species in Australia [*M*. *caligula* (Debenham, 1987)] and four named fossil species from Eocene Baltic amber (*M*. *wirthi* Szadziewski, 1988; *M*. *gedanicola* Szadziewski, 1988; *M*. *nielseni* Szadziewski, 1988; *M*. *miocaenica* Szadziewski, 1993) have been described.

*Meunierohelea cambayana* Stebner & Szadziewski n. sp.

urn:lsid:zoobank.org:act:7F551482-1B19-4019-920E-50A45F31905E

**Fig 4:**
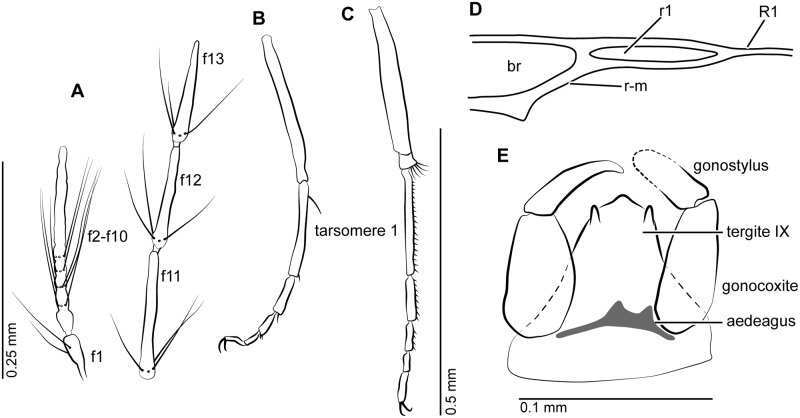
*Meunierohelea cambayana* n. sp. from early Eocene Cambay amber, holotype male, no. Tad-507. **A**. Antenna, flagellomeres (f1-f13). **B**. Tibia and tarsus of mid leg. **C**. Tibia and tarsus of hind leg. **D**. Part of radial sector of wing. **E**. Male genitalia, ventral view.

**Fig 5:**
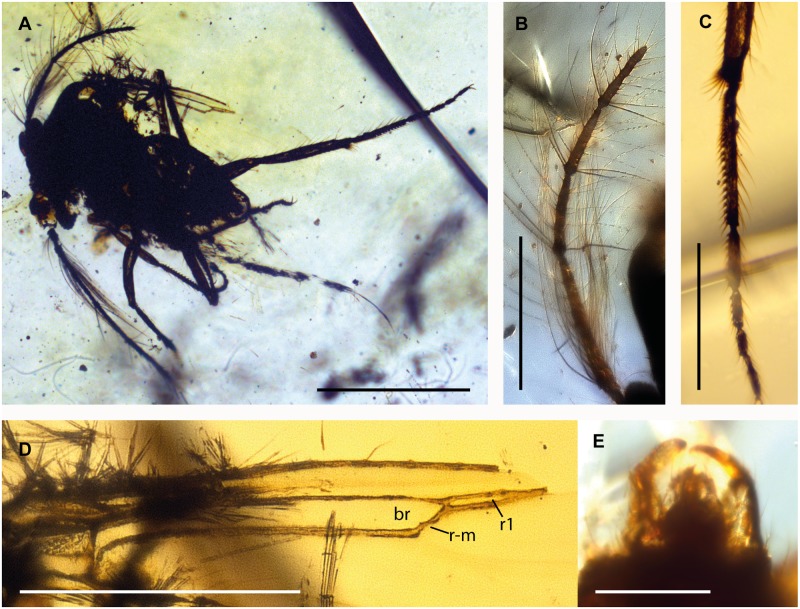
*Meunierohelea cambayana* n. sp. from early Eocene Cambay amber, holotype male, no. Tad-507. **A**. Habitus. **B**. Flagellum. **C**. Tibia and tarsus of hind leg. **D**. Radial sector of wing with cell r_1_. **E**. Male genitalia, dorsal view. Scale bars A: 0.5 mm, B-D: 0.2 mm, E: 0.1 mm.

Figs 4A–4E and 5A–5E

#### Type material

Holotype male: No. Tad-507, in amber from India: Gujarat, Tadkeshwar lignite mine, Cambay Formation (lower Eocene, Ypresian); in a box labelled “*Meunierohelea cambayana* Stebner & Szadziewski. **HOLOTYPE** AMNH IN-Tad-507. Cambay Form. Gujarat India”. Other material. Male, Tad-519, is poorly preserved, doubtfully determined and therefore not considered a paratype.

#### Diagnosis

Male: only species of *Meunierohelea* with AR 1.4, cell r_1_ well developed, and a slender gonostylus.

#### Description

Male. Body length 0.8 mm.

*Head*. Flagellum (Fig 4A) with flagellomeres 2–10 fused, length: 0.70 mm; AR 1.4; plume well developed. Proboscis short. Palpus with 5 segments.

*Wing*. Poorly preserved, slender, length 0.63 mm. cell r_1_ well-developed; cell r_2_ not preserved (Fig 4D). Wing membrane covered with distinct microtrichia, macrotrichia absent.

*Legs* (Fig 4B and 4C). Femur, tibia of hind leg slightly stouter than those of other legs; fourth tarsomeres cylindrical; tarsomeres 1–3 of hind leg with palisade setae, tibial comb with 5 spines; TR (II) approximately 2.2, TR(III) 2.4.

*Genitalia* (Fig 4E). Inverted; gonocoxite not modified; gonostylus slender, tapering evenly to acute apex, slightly bent; one gonostylus swollen, with broadly rounded apex which is most likely an artefact. Parameres not visible.

Female unknown.

#### Etymology

The specific name refers to the Cambay Formation.

#### Discussion

The fused flagellomeres 2–10 and a gonostylus tapering to its apex in *Meunierohelea cambayana* n. sp. resembles those of *M*. *gedanicola* and *M*. *nielseni* from Baltic amber. *Meunierohelea cambayana* n. sp. can be distinguished from the two Baltic amber species by its AR, which is distinctly lower in *M*. *nielseni* and *M*. *gedanicola* (below 1.0).

*Meunierohelea borkenti* Stebner & Szadziewski n. sp.

urn:lsid:zoobank.org:act:D6E21814-FDA5-42B4-A8A3-45365C1D0B4E

**Fig 6:**
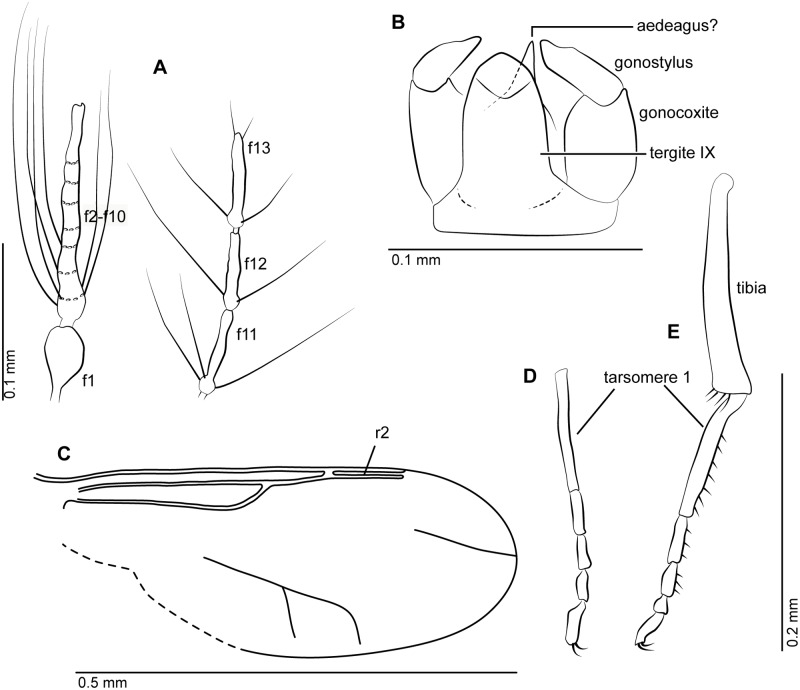
*Meunierohelea borkenti* n. sp. from early Eocene Cambay amber, holotype male, no. Tad-516. **A**. Antenna, flagellomeres (f1-f13). **B**. Genitalia, dorsal view. **C**. Wing. **D**. Tarsus of mid leg. **E**. Tibia and tarsus of hind leg.

**Fig 7:**
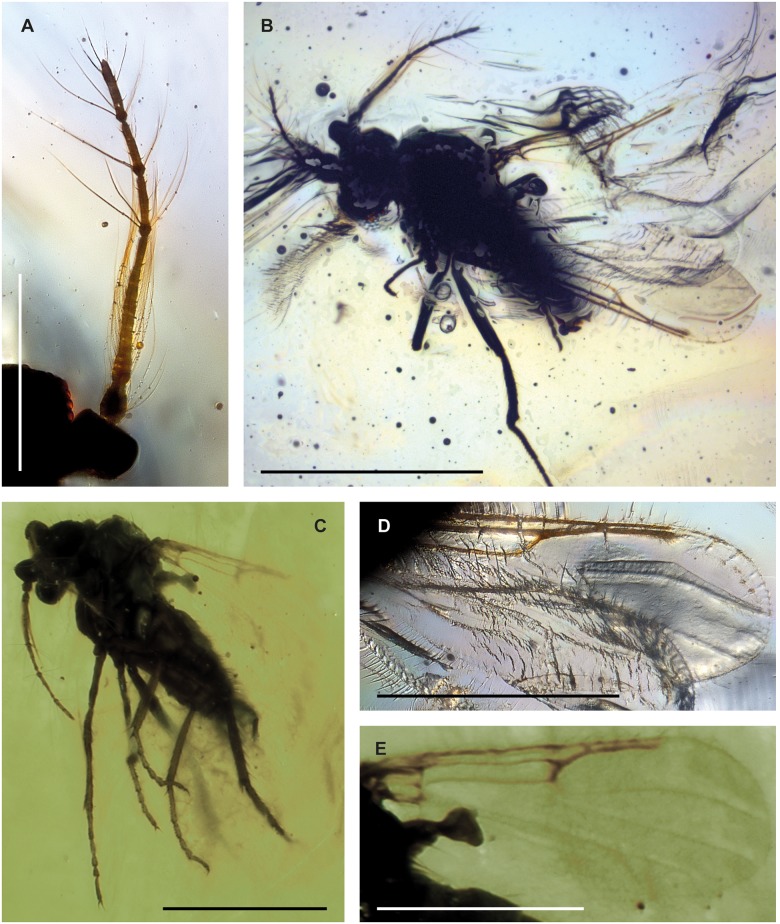
Males of *Meunierohelea borkenti* n. sp. (holotype, Tad-516) and *Meunierohelea orientalis* n. sp. (holotype, Tad-858 a) from early Eocene Cambay amber. **A**. *M*. *borkenti* n. sp., antenna. **B**. *M*. *borkenti* n. sp., habitus. **C**. *M*. *orientalis* n. sp., habitus **D**. *M*. *borkenti* n. sp., wing. **E**. *M*. *orientalis* n. sp., wing. Scale bars A: 0.2 mm, B, C: 0.5 mm, D, E: 0.3 mm.

Figs 6A–6E and 7A, 7B and 7D

#### Type material

Holotype male: No. Tad-516, in amber from India: Gujarat, Tadkeshwar lignite mine, Cambay Formation (lower Eocene, Ypresian); in a box labelled “*Meunierohelea borkenti* Stebner & Szadziewski. **HOLOTYPE** AMNH IN-Tad-516. Cambay Form. Gujarat India”.

#### Diagnosis

Male: only species of *Meunierohelea* with cell r_1_ absent, AR slightly lower than 1.0, and a broad gonostylus.

#### Description

Male. Body length 0.7 mm.

*Head*. Flagellum (Fig 6A) with flagellomeres 2–10 fused, length 0.37 mm, AR 0.9; plume well developed. Proboscis short. Palpus with 5 segments.

*Wing*. Slender; cell r_1_ absent, cell r_2_ with opened apex (Fig 6C). Length approximately 0.51 mm; CR 0.80. Wing membrane without macrotrichia.

*Legs* (Fig 6D and 6E) slender, fourth tarsomeres cylindrical; tarsomeres 1–3 of hind leg with palisade setae, TR (II) 2.7, TR(III) 2.8.

*Genitalia*. Gonostylus broad, cylindrical, abruptly tapering at distal 5^th^, ending in blunt apex (Fig 6B). Pointed structure below tergite IX might be aedeagus, parameres not visible.

Female unknown.

#### Etymology

Dedicated to Art Borkent in recognition of his knowledge and valuable contributions to the study of biting midges.

#### Discussion

*Meunierohelea borkenti* n. sp. can be distinguished from all species of the genus by the absence of cell r_1_ and a broad gonostylus.

*Meunierohelea orientalis* Stebner & Szadziewski n. sp.

urn:lsid:zoobank.org:act:EB808952-EAFB-4BD0-A109-889CB02E554E

**Fig 8:**
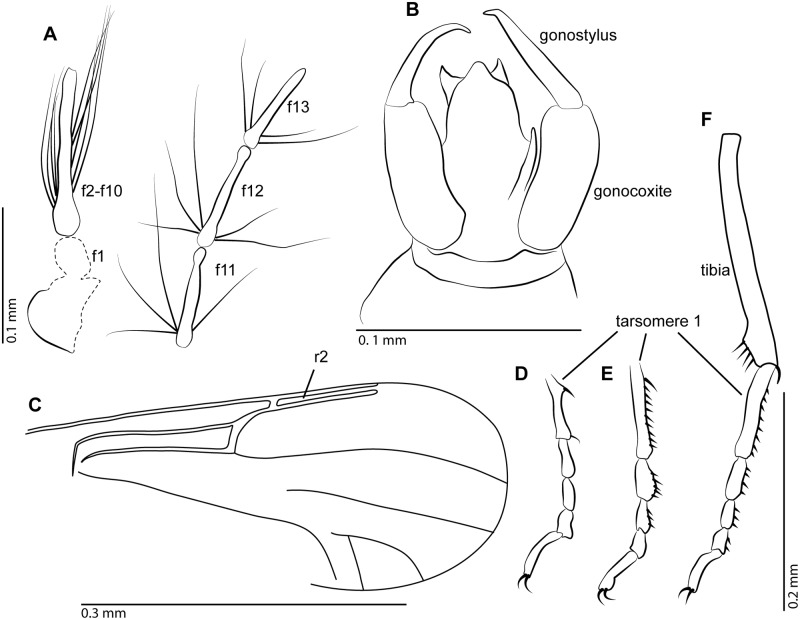
*Meunierohelea orientalis* n. sp. from early Eocene Cambay amber, holotype male, no. Tad-858a. **A**. Antenna, pedicel and flagellomeres (f1-f13). **B**. Genitalia, ventral view. **C**. Wing. **D**. Tarsus of fore leg. **E**. Tarsus of mid leg. **F**. Tibia and tarsus of hind leg.

Figs 7C and 7E and 8A–8F

#### Type material

Holotype male: No. Tad-858 a, in amber from India: Gujarat, Tadkeshwar lignite mine, Cambay Formation (lower Eocene, Ypresian); in a box labelled “*Meunierohelea orientalis* Stebner & Szadziewski. **HOLOTYPE** AMNH IN-Tad-858a. Cambay Form. Gujarat India”.

#### Diagnosis

Male: the only species of *Meunierohelea* with cell r_1_ absent, and a long slender gonostylus.

#### Description

Male. Complete, dark, generally opaque due to preservation. Body length 0.75 mm.

*Head*. Flagellum (Fig 8A) with flagellomeres 2–10 fused, length: 0.37 mm, AR 1.25; plume well developed. Proboscis, palpus not visible.

*Wing* (Fig 8C). Slender, cell r_1_ absent, cell r_2_ with opened apex. Length 0.40 mm; CR 0.70. Wing membrane without macrotrichia.

*Legs* (Fig 8D–8F). Hind leg stout, fourth tarsomeres cylindrical; tarsomere 1 of fore leg with one strong, curved subbasal, one subapical spine, tarsomere 1 of mid leg with subbasal spine, tarsomeres 1–3 of mid-, hind legs with palisade setae, tibial comb with 4 spines, all claws with subbasal inner tooth, TR(I) 2.2, TR(II) 2.8, TR(III) 2.4.

*Genitalia* (Fig 8B). Gonocoxite, gonostylus long, slender; gonostylus slightly bent, evenly tapering to pointed apex; parameres, aedeagus barely visible.

Female unknown.

#### Etymology

The specific name refers to the Oriental Region.

#### Discussion

*Meunierohelea orientalis* n. sp. can be easily distinguished from all Baltic amber species by the presence of a strong curved subapical spine on tarsomere 1 of the fore leg and by the absence of the first radial cell. The species can be distinguished from *M*. *borkenti*, which also is missing cell r_1_, by a higher antennal ratio and a longer and slender gonostylus.

## 4. Discussion

### 4.1 Palaeohabitat

According to Borkent [28] the proportion of extant male to female Ceratopogonidae within their habitat and summed as a group, is about 40:60 and shifts with distance from the original habitat in favor of the females because of female dispersal. In Cambay amber a proportion of 39:61 in favor of the females indicates entrapment at the site of emergence. This indicates that Ceratopogonidae based habitat reconstruction can be applied directly to the resin producing forest.

Ceratopogonidae in Indian Cambay amber include representatives, such as *Leptoconops*, which fed on vertebrate blood as well as taxa that fed on the liquified contents of other insects (resulting from injected proteolytic saliva). Females of *Meunierohelea*, *Serromyia*, *Eohelea*, *Mantohelea*, *Gedanohelea*, *Camptopterohelea* and *Stilobezzia* (all in the tribe Ceratopogonini) were predators of male insects of similar or smaller size such as Chironomidae, Chaoboridae or Ceratopogonidae (Table 2). For *Eohelea* [45, 46] and *Serromyia* [47] it has been shown that females also fed on the males during mating. Adults of *Forcipomyia* and most probably also *Stilobezzia* are important pollinators for a number of trees and plants [48]. Larval ecology is known for some of the modern taxa recorded from Cambay amber and ranges from mainly aquatic to terrestrial habitats (Table 2). *Forcipomyia* larvae and pupae can be found in a variety of terrestrial to aquatic habitats where they feed on algae and rotting plants. *Brachypogon* larvae live in aquatic and semi-aquatic habitats and can be found in small ponds, at the edges of lakes and rivers and in wet turf [49]. Species of *Stilobezzia* inhabit a variety of aquatic habitats like lakes, streams, rivers, ponds, swamps and marshes, tree hollows and other phytotelmata [49].

**Table 2:** Ecology of female adults and larvae of Ceratopogonidae genera found in Cambay amber.

Genus	Adult ♀	Larvae
*Leptoconops*	blood feeders on vertebrates	in saturated sands, soils at sea shores, estuaries or deserts
*Forcipomyia*	blood feeders on vertebrates, predators of insects	under the bark of trees, in rotting roots, mosses, mat of algae, wet soil, phytotelmata
*Brachypogon*	predators of insects	in ponds, edges of lakes and rivers, humid turf
*Camptopterohelea*	predators of insects	as in other extant Ceratopogonini, larvae are probably semiaquatic or aquatic
†*Eohelea*	predators of insects	as in other extant Ceratopogonini, larvae were probably semiaquatic or aquatic
†*Gedanohelea*	predators of insects	as in other extant Ceratopogonini, larvae were probably semiaquatic or aquatic
†*Mantohelea*	predators of insects	as in other extant Ceratopogonini, larvae were probably semiaquatic or aquatic
*Meunierohelea*	predators of insects	as in other Ceratopogonini, larvae are probably semiaquatic or aquatic
*Serromyia*	predators of insects	in mosses at lake margins, in mud associated with marshlands
*Stilobezzia*	predators of insects	in lakes, streams, rivers, ponds, swamps, marshes, tree hollows, phytotelmata

^†^ fossil taxa

*Leptoconops* species are generally associated with xeric habitats and larvae develop in wet alkaline or saline sand at sea shores, estuaries or deserts today. *Serromyia* species are associated with bogs, fens, wet meadows, streams or small rivers [50]. Their larvae occur in mosses at lake margins and in mud associated with marshlands [50, 49].

Ceratopogonidae-based reconstruction of the palaeoenvironment primarily depicts a forest growing under very humid conditions. Most modern representatives of the genera recorded from Cambay amber have aquatic to semi-aquatic larvae, indicating a moist habitat rich in decomposing plant material with permanent aquatic habitats like marshes or bogs, and some temporary waters like puddles, or water loaded tree hollows. Findings of *Leptoconops* prove a near shore resin production which corresponds with sedimentological analyses that interpreted the depositional system as a low energy near shore/coastal environment, ranging through lacustrine, swampy, marshy and deltaic environments [51]. The accompanying fauna must have been rich in small insects like Chironomidae, which are in fact the most common dipteran group in Indian amber. Furthermore, from Vastan and Tadkeshwar lignite mine bats [52], birds [53, 54], lizards [55, 56], and a number of mammals (summarized in [16, 57]) have been recorded which could have served as potential hosts for *Leptoconops* [58].

### 4.2 Biogeography

Knowledge of present day’s diversity is far from being complete, especially in megadiverse areas, such as the Neotropics or Australasia. Likewise, the knowledge of fossil insects still is fragmentary. This is partly due to the lack of deposits, but also to the fact that the fossil record itself does not display the actual past diversity. Nevertheless, there are constantly new fossil deposits being discovered from all over the world, providing more and more information about past diversity and allowing analysis of biogeographic patterns. In this context, Cambay amber as well as Fushun amber are of great significance because together with Oise amber from France they fill a large gap in the spatial fossil record of the Paleogene.

**Fig 9:**
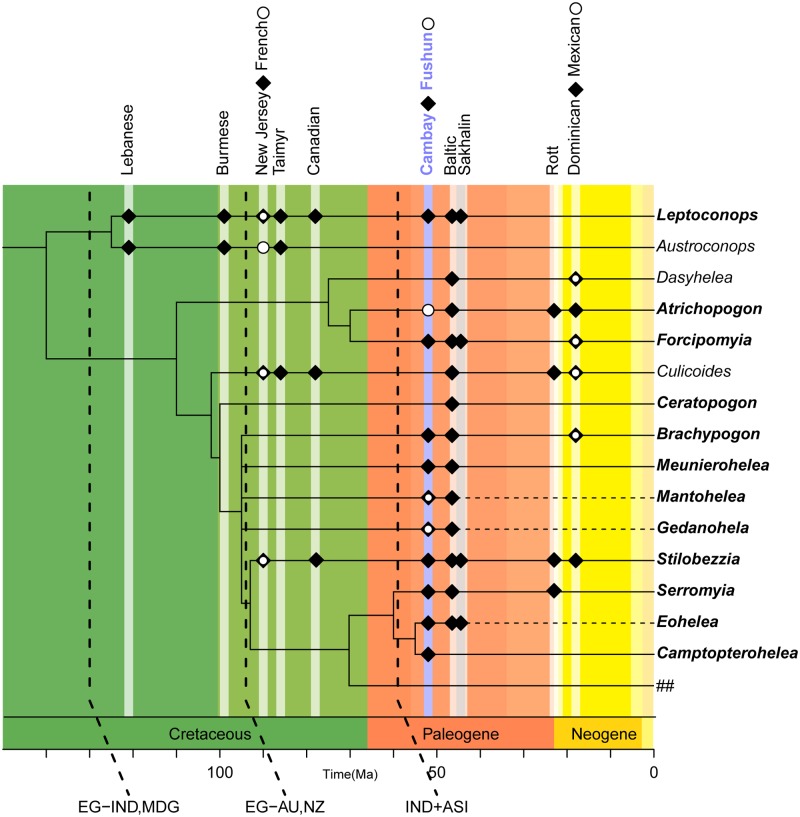
Relationships of select extant and fossil Ceratopogonidae genera (compiled from data after [28, 59, 60, 63]). Dashed lines in the cladogram refer to extinct taxa, solid lines to extant taxa. ## refers to genera in tribes different than Ceratopogonini (Heteromyiini, Hebetulini, Johannsenomyiini, Sphaeromiini, Palpomyiini and Stenoxenini).

Beckenbach and Borkent [59] proposed a phylogenetic relationship for 14 species from 12 genera of Ceratopogonidae based on mitochondrial cytochrome oxidase subunit 2 as well as morphological characters. Previously, Borkent [28] demonstrated that the fossil record has a high congruence with the cladistic results (meaning that earlier fossils represent only older lineages). Incorporation of the taxa found in Cambay and Fushun amber clearly supports this finding (Fig 9).

The earliest extant lineage of Ceratopogonidae are the Leptoconopinae with two extant genera, *Leptoconops* (worldwide) and *Austroconops* (now restricted to southwest Australia). Both of these genera have been recorded in Lower Cretaceous deposits. Only two fossils of *Leptoconops* are recorded from Cambay amber and no fossils have been found in Fushun amber so far. Another early clade of biting midges, the Forcipomyiinae (including *Forcipomyia* and *Atrichopogon*), is represented by 12 fossils from one genus in Cambay amber (*Forcipomyia*) and five specimens in Fushun amber (*Atrichopogon +* Forcipomyiinae indet., pers. observ.) respectively.

Seven out of nine genera recorded from Cambay amber belong to the tribe Ceratopogonini (all genera from *Ceratopogon* to *Camptopterohelea* in Fig 9), a paraphyletic group whose relationships are not yet fully understood although a comprehensive analysis of pupal data contributed to partially resolving phylogenetic relationships [60]. The oldest Ceratopogonini are recorded from Cretaceous New Jersey, French and Canadian ambers followed by representatives in early Eocene Cambay (54 Ma) and Fushun amber (53 Ma) (herein and [43]). It is, to some extent, striking that most Ceratopogonini as well as the Forcipomyiinae in Cambay and Fushun amber represent their first appearances in the fossil record. This includes the extant genera *Brachypogon*, *Serromyia*, *Meunierohelea*, *Forcipomyia* and *Atrichopogon*, as well as the extinct taxa *Gedanohelea*, *Eohelea* and *Mantohelea*, and the first fossil of the modern genus *Camptopterohelea*. Besides the fact that the late Paleocene-middle Eocene was one of the hottest periods of the Cenozoic, which probably triggered radiation of many plant and animal taxa (e.g. [61]), fossils in Cambay amber might also provide evidence for the theory that species diversification is strongly linked to geodynamic activity. The Indian-Asian collision, which finally led to the uplift of the Himalaya, certainly was a major tectonic event that resulted in habitat fragmentation and formation of heterogeneous habitats. It has been shown that the Andean uplift for example was the leading event for the evolution of the Amazonian current biodiversity [62] and the same mechanism might be true for the regions adjacent to the collision boundary of India and Asia.

Explanations: EG-IND, MDG = beginning of separation of India (IND) and Madagascar (MDG) from East Gondwanaland (EG) (after [64]); EG-AU, NZ = beginning of separation of Australia (AU) and New Zealand (NZ) from East Gondwana (after [64]); IND+ASI = collision of India with Asia (after [65]).

Distribution patterns of extant and extinct representatives of the fossils recorded from Cambay amber (Table 3) show that amber from India includes:

- extant genera that have a nearly global distribution today and are also known from geographically and chronologically distinct amber deposits (*Leptoconops*, *Forcipomyia*, *Atrichopogon*, *Brachypogon*, *Stilobezzia*) (e.g. [28, 59, 66]).
- extant taxa that have limited distributional patterns today. The genus *Meunierohelea* has been recorded from amber of the Baltic Region [32, 44, 66] and with one extant species from modern Australia [67]. A similar relictual distribution can be observed in the extant genus *Metahelea* [66]. *Camptopterohelea* is known from only five extant species, which have exclusively been found in the Oriental Region (India, Indonesia, Philippines, and Malaysia) [68, 69, 70].
- fossil taxa which were distributed during the Paleogene in Europe and Asia only. *Eohelea* is known from Eocene Sakhalin, Baltic and Cambay amber and *Gedanohelea*, previously known from Baltic amber, has recently also been recorded from Fushun amber [43] and from Cambay amber (present records). The extinct genus *Mantohelea* has been reported from Baltic (2 species), Fushun (1 species) and Cambay (1 specimen, present record) amber [32, 43].

**Table 3:** Distribution of select extant and fossil Ceratopogonidae genera (data after [2,5]). Abbreviations: Ne: Nearctic Region; Nt: Neotropic Region; Pal: Palaearctic Region; Af: Afrotropical Region; OR: Oriental Region; Aus: Australia; Mad: Madagascar; NZ: New Zealand; Ca: Cambay amber; Fu: Fushun amber; Ba: Baltic amber.

	Distribution										
	Recent								Eocene amber		
Genus	Ne	Nt	Pal	Af	OR	Aus	Mad	NZ	Ca	Fu	Ba
*Leptoconops*	x	x	x	x	x	x	x		x		x
*Forcipomyia*	x	x	x	x	x	x	x	x	x	x	x
*Atrichopogon*	x	x	x	x	x	x	x	x		x	x
*Brachypogon*	x	x	x	x	x	x			x		x
*Stilobezzia*	x	x	x	x	x	x	x	x	x		x
*Meunierohelea*						x			x		x
*Camptopterohelea*					x				x		
*Serromyia*	x		x	x	x	x	x		x		x
†*Mantohelea*									x	x	x
†*Eohelea*									x		x
†*Gedanohelea*									x	x	x

^†^ fossil taxa

**Fig 10:**
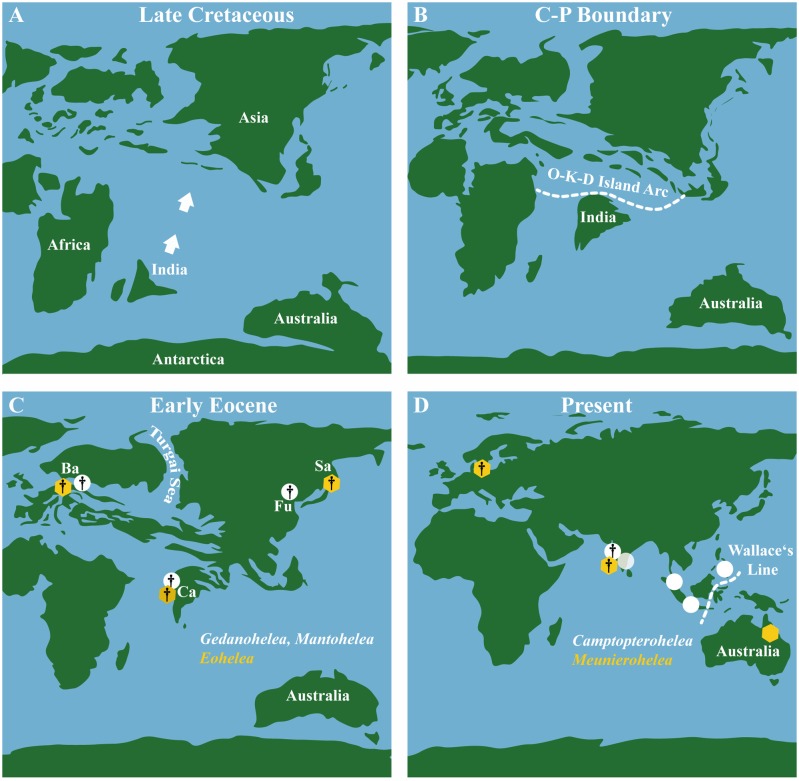
Plate tectonic history of the Indian subcontinent from the Late Cretaceous to Present and distribution of select biting midge taxa (maps modified after data supplied by “Global Paleogeography and Tectonics in Deep Time 2016 Colorado Plateau Geosystems Inc.”). **A**. Late Cretaceous. **B**. Cretaceous-Paleogene Boundary. Dashed lines showing possible dispersal routes between India and Africa/Asia (Oman-Kohistan-Dras Island Arc, summarized in Chatterjee & Scotese [5]). **C**. Early Eocene. Circles and hexagon showing fossil (†) genera of *Gedanohelea*, *Mantohelea* and *Eohelea* in Eocene ambers. Abbreviations: Ba = amber from the Baltic Region, Ca = Cambay amber, Fu = Fushun amber, Sa = Sakhalin amber. **D**. Present. Circles and hexagon showing fossil (†) genera in Eocene ambers and recent representatives of *Meunierohelea* and *Camptopterohelea*.

This mixture of different faunal links demonstrates that, at the generic level, the biting midge fauna from Cambay amber was not endemic to India during the Eocene. Instead, Ceratopogonini reveal affinities to slightly younger amber from the Baltic region and contemporaneous Fushun amber from eastern Asia (Fig 10C) as well as to modern faunas from Australia and the Oriental Region (Fig 10D). Based on the occurrence of the earliest fossils, the origin of Ceratopogonini has been estimated to Late Cretaceous age (90 Ma [59, 28]). At that time the Indian subcontinent, which started separating from East Gondwanaland (Antarctica, Australia, New Zealand, India) in the Early Cretaceous (ca 130 Ma), was already on its drift northwards (e.g. [64]) (Fig 9, Fig 10A and 10B). The estimated age of the fossils (*Gedanohelea*, *Eohelea*, *Camptopterohelea*, *Mantohelea*) and their distributional patterns (i.e. present only in Europe and Asia during the Paleogene) (Fig 10C) together with India’s geological history implies that they are not of Gondwanan origin but that faunal exchange between India and Asia/Europe occurred before the formation of the amber in the early Eocene and that dispersal was one important factor that shaped India’s biota at that time.

Whether this exchange took place by transoceanic dispersal or geodispersal (expansion of species when geographical barriers disappear) remains unsolved for now. The latter scenario however might be explained by land bridge connections between drifting India and Asia (Oman-Kohistan-Dras Island Arc, Fig 10B, summarized in [5]) or by a collision of the Indian subcontinent with Asia prior to the time of amber formation at 54 Ma. A late Paleocene collision date (59 Ma) has recently been supported by the study of radiolarian and nannofossil biostratigraphy and detrital zircon geochronology [65]. Nevertheless, the Oman-Kohistan-Dras Island Arc, which existed during the latest Cretaceous between India, Africa and Asia (Fig 10B), could have acted as a geodispersal route for Ceratopogonidae, as has been suggested for Maastrichtian tetrapods [5].

The distribution of *Meunierohelea* with only one extant species known from Australia but four named fossil species in European Baltic amber [32] and three in Indian Cambay amber (present paper) might indicate that this genus has had a much broader distribution in the past than in present times (Fig 10D). Nevertheless it has to be considered that the modern Oriental Region, including India, is poorly sampled and might therefore harbor undiscovered species of *Meunierohelea* that dispersed to Australia only recently. In this context, knowledge about the phylogenetic relationships of the fossil and extant species of *Meunierohelea* would help in resolving their historical zoogeography; i.e. if the extant species is the sister group to all fossil species or if it is more closely related to one fossil species.

In contrast to the aforementioned taxa, fossil as well as recent *Camptopterohelea* show a very limited occurrence restricted to the Oriental Region (Fig 10D). Borkent & Picado [71] proposed a sister group relationship between *Cacaohelea*+*Parastilobezzia* and *Camptopterohelea*+*Eohelea*. Since the latter two taxa are now recorded from the early Eocene they must have diverged from the *Cacaohelea*+*Parastilobezzia* assemblage and from each other prior to this time. The *Cacaohelea*+*Parastilobezzia* group is restricted to the Neotropical Region today, whereas the *Camptopterohelea*+*Eohelea* lineage has been recorded from the Palaearctic and Oriental regions only. This distribution pattern suggests that the two groups (New World and Old World group) might have been separated by a vicariance event and, furthermore, that *Camptopterohelea* and *Eohelea* originated and diversified in the Palaearctic/Oriental region. Distribution of extant species of *Camptopterohelea* exactly follows the northern boundary of Wallace’s Line (Fig 10D), which separates the fauna of Asia and Australia (although some authors consider the Philippines, which harbor one species of *Camptopterohelea*, to belong to the transitional area “Wallacea” between the two biogeographic provinces). Faunal exchange of Asian biota with the islands of the Malay Archipelago northern of Wallace’s Line was facilitated during the Pleistocene glaciations when, due to sea level decline, Asia was united with these islands on its continental shelves (e.g. [72]). Distribution of *Camptopterohelea* might indicate that dispersal of this taxon is restricted by sea, which in turn might imply that this genus entered (or dispersed from) India before the early Eocene via geodispersal rather than by transoceanic dispersal. However, it has to be considered that parts of the archipelago between mainland Southeast Asia and Australia, like New Guinea for example, are poorly collected and that *Camptopterohelea* might be present on one of the islands southern of the Philippines.

Certainly, all these hypotheses strongly depend on factors related to the quality of the fossil record, including the rather small sample size available for the present work, and to the state of knowledge about Ceratopogonidae phylogeny. Further resolution of relationships within Ceratopogonidae, both below and above genus level, would help in understanding and interpreting past and present distribution patterns in order to contribute to reconstructing India’s plate tectonics history. In this context the scarcity of known fossil deposits in Africa, as well as the lack of phylogenetic information about modern Ceratopogonini in Africa, is a major handicap. It is thus possible, that the so far missing African connections of Cambay biting midges (as in many other insects groups found in Indian amber), which could be evidence for Gondwanan distribution of the respective clades, simply is a result of lack of knowledge. Similarly, there are no significant Paleocene and Eocene amber deposits in the New World, which hampers the analysis of palaeobiogeographic patterns, because there is no information about whether the distribution of Old World fossils extended to the Nearctic or not at that time. Thus, absence of a group from the fossil record does not necessarily mean that the taxon in question was not present at that time but simply that it has not been sampled or preserved. Hence, there is a reasonable possibility that some taxa such as *Culicoides*, an earlier lineage of Ceratopogoninae and well known from various deposits since the Cretaceous, will be discovered in Indian amber in the future.

For helping to understand India´s plate tectonics history the phylogenetic relationships as well as the age of origination of the various clades, and also the knowledge of Asian faunas subsequent to Cambay amber, which is rather fragmentary, are of great importance. In this regard future studies on insects in middle Miocene Zhangpu amber from Southeast China might also prove to be of great interest [73].

Despite the uncertainties discussed above and the fact that the fossil assemblage studied in the present work is rather small, data recorded here display a valuable source of information for Indian amber research, which is still in its beginning, and should be regarded as a small fraction of a puzzle that still is far from being complete.

## Supporting Information

S1 Fig *Brachypogon* and *Forcipomyia* fossils from early Eocene Cambay amber. **A**. Tad-508 *Brachypogon* sp.♀. **B**. Tad-854 *Brachypogon* sp. ♀. **C**. Tad-851 *Brachypogon* sp. ♂. **D**. Tad-163 *Forcipomyia* sp. ♀. **E**. Tad-565 *Forcipomyia* sp. ♀. **F**. Tad-602 *Forcipomyia* sp. ♀. **G**. Val-3.4 *Forcipomyia* sp. ♂. **H**. Tad-860 *Forcipomyia* sp. ♂. Scale bars A, B, D-H: 0.2 mm, C: 0.1 mm.
(TIF)

S2 Fig *Gedanohelea*, *Stilobezzia*, *Leptoconops* and *Mantohelea* fossils from early Eocene Cambay amber. **A**. Val-3.2 *Gedanohelea gerdesorum* n. sp., paratype ♀. **B**. Tad-853a *Stilobezzia* sp. ♀. **C**. Tad-506 *Leptoconops* sp. ♂. **D**. Tad-673 *Mantohelea* sp. ♀. Scale bars A, C, D: 0.2 mm, B: 0.5 mm.
(TIF)

S1 Table Syninclusions of the samples investigated in the present study. (DOCX)
