# Correction: Biting Midges (Diptera: Ceratopogonidae) from Cambay Amber Indicate that the Eocene Fauna of the Indian Subcontinent Was Not Isolated

**DOI:** 10.1371/journal.pone.0173135

**Published:** 2017-03-29

**Authors:** The PLOS ONE Staff

There is an error in the penultimate sentence of the first paragraph of the Materials and Methods. The correct sentence is: All specimens will be deposited in the collection of the Birbal Sahni Institute of Palaeosciences, Lucknow, India

A portion of the figure legend for Fig 9 is incorrectly displayed in the fifth paragraph under the subheading “Biogeography” in the Discussion section. The publisher apologizes for the error. Please see the complete, correct Fig 9 caption here.

**Fig 9:**
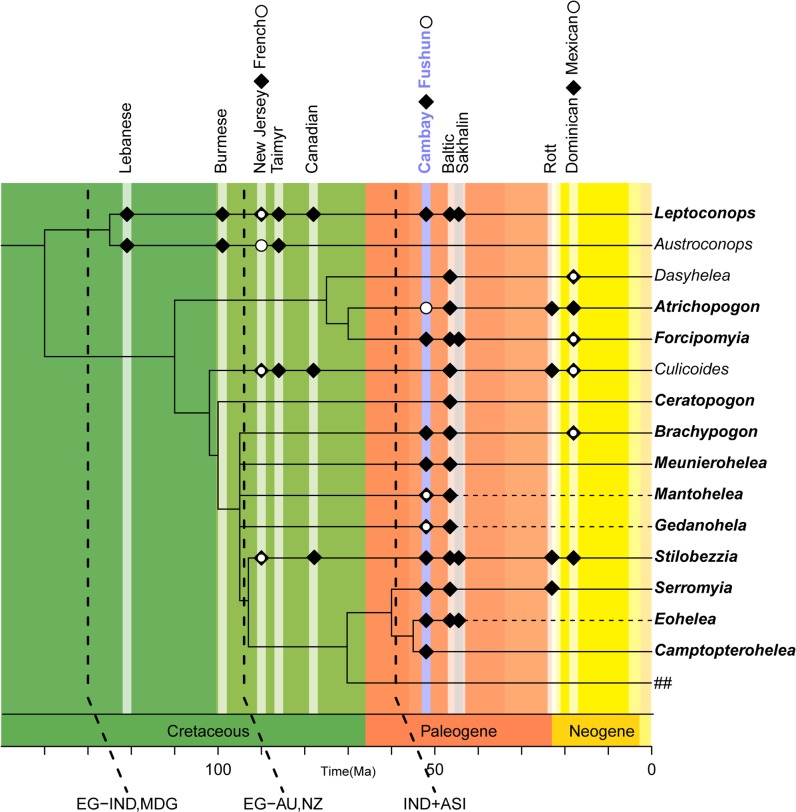
Relationships of select extant and fossil Ceratopogonidae genera (compiled from data after [28, 59, 60, 63]). Dashed lines in the cladogram refer to extinct taxa, solid lines to extant taxa. ## refers to genera in tribes different than Ceratopogonini (Heteromyiini, Hebetulini, Johannsenomyiini, Sphaeromiini, Palpomyiini and Stenoxenini). Explanations: EG-IND, MDG = beginning of separation of India (IND) and Madagascar (MDG) from East Gondwanaland (EG) (after [64]); EG-AU, NZ = beginning of separation of Australia (AU) and New Zealand (NZ) from East Gondwana (after [64]); IND+ASI = collision of India with Asia (after [65]).
