# Supplementary figures and images for: Biting Midges (Diptera: Ceratopogonidae) from Cambay Amber Indicate that the Eocene Fauna of the Indian Subcontinent Was Not Isolated
Source: PLoS One. 2017 Jan 11;12(1):e0169144. doi: 10.1371/journal.pone.0169144 (PMC5226682; doi:10.1371/journal.pone.0169144)

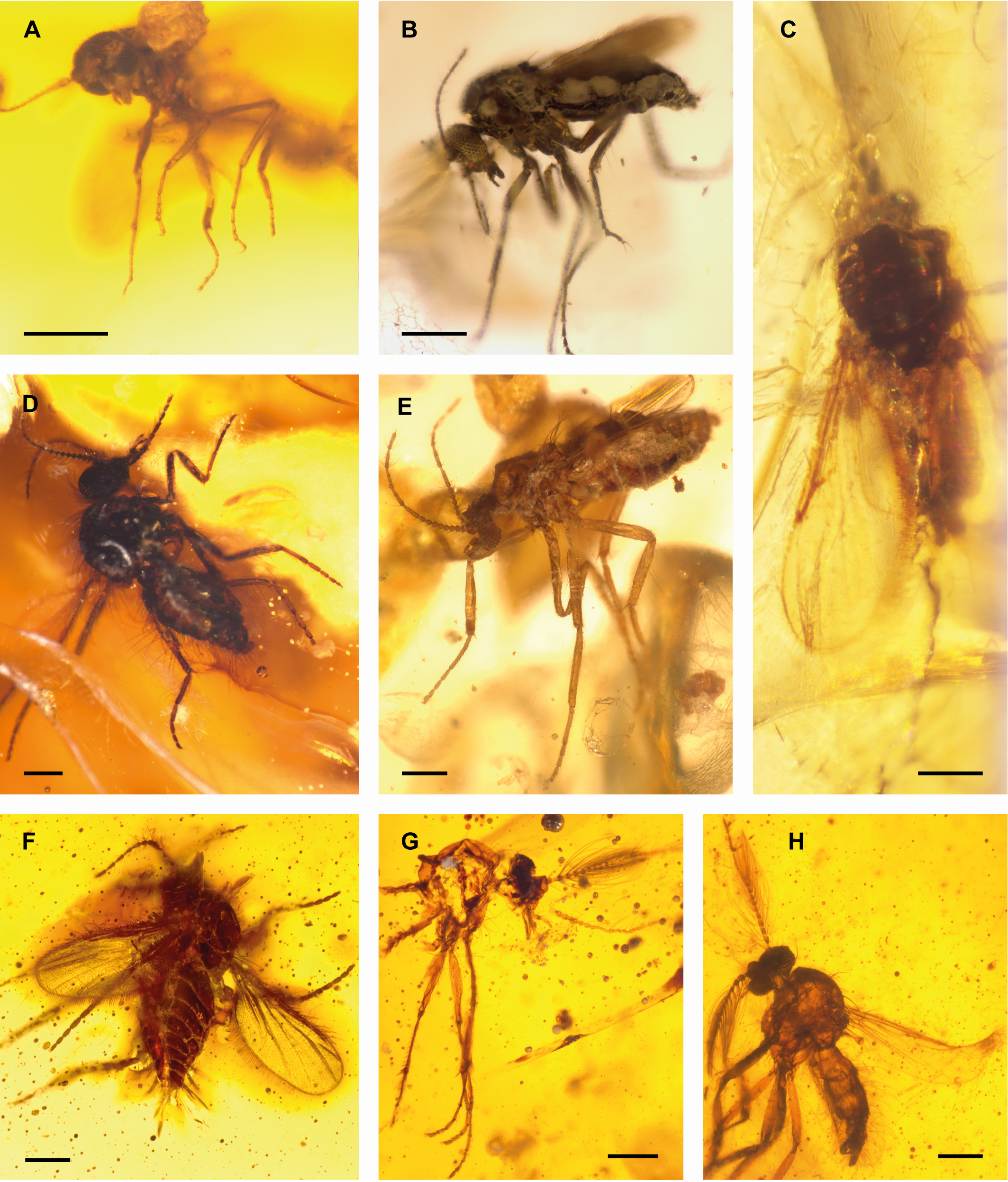

Supplement: S1 Fig — A. Tad-508 Brachypogon sp.♀. B. Tad-854 Brachypogon sp. ♀. C. Tad-851 Brachypogon sp. ♂. D. Tad-163 Forcipomyia sp. ♀. E. Tad-565 Forcipomyia sp. ♀. F. Tad-602 Forcipomyia sp. ♀. G. Val-3.4 Forcipomyia sp. ♂. H. Tad-860 Forcipomyia sp. ♂. Scale bars A, B, D-H: 0.2 mm, C: 0.1 mm. (TIF) [file pone.0169144.s001.tif]

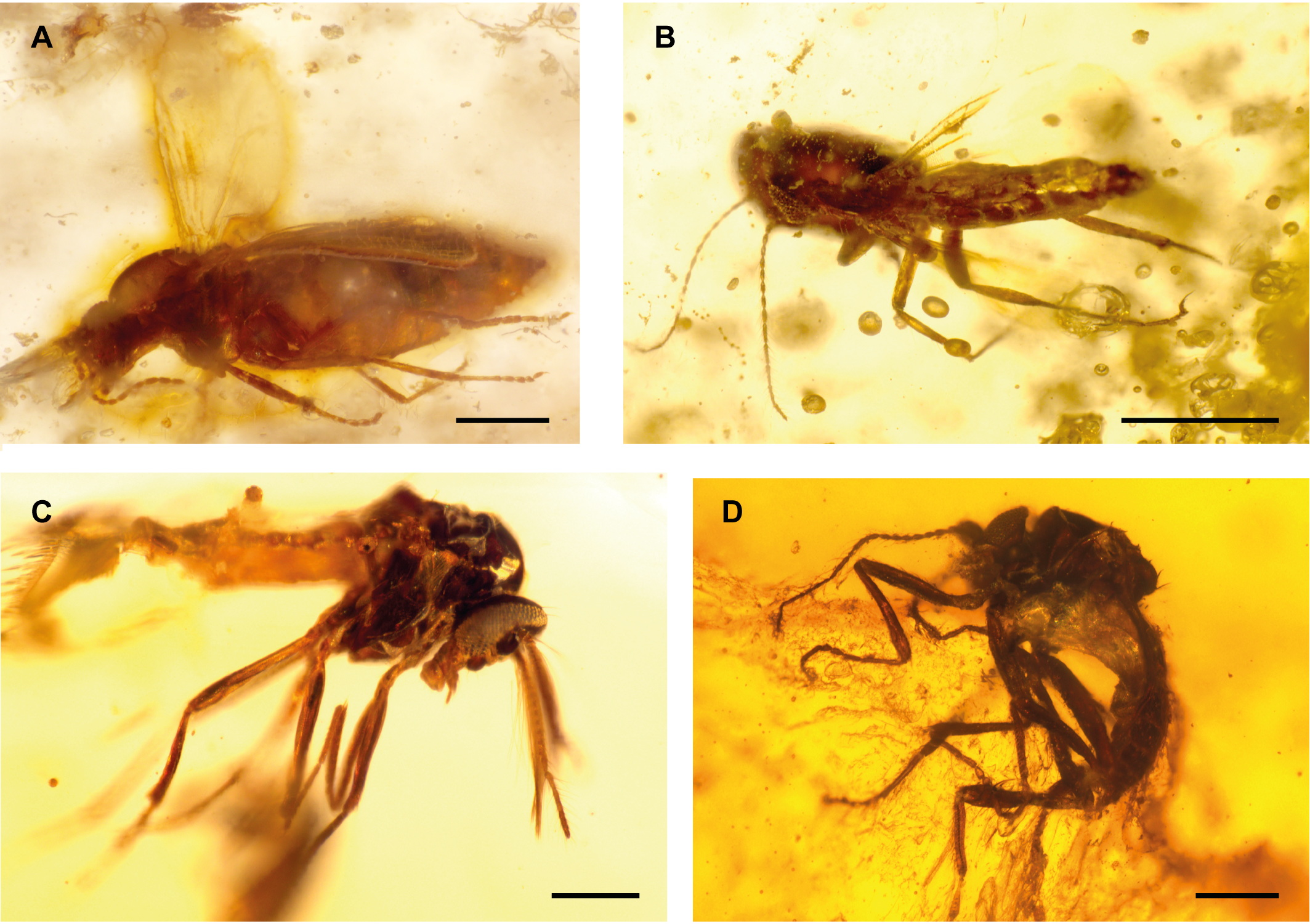

Supplement: S2 Fig — A. Val-3.2 Gedanohelea gerdesorum n. sp., paratype ♀. B. Tad-853a Stilobezzia sp. ♀. C. Tad-506 Leptoconops sp. ♂. D. Tad-673 Mantohelea sp. ♀. Scale bars A, C, D: 0.2 mm, B: 0.5 mm. (TIF) [file pone.0169144.s002.tif]
