# Supplementary material for: Biting Midges (Diptera: Ceratopogonidae) from Cambay Amber Indicate that the Eocene Fauna of the Indian Subcontinent Was Not Isolated
Source: PLoS One. 2017 Jan 11;12(1):e0169144. doi: 10.1371/journal.pone.0169144 (PMC5226682; doi:10.1371/journal.pone.0169144)
Supplement: S1 Table — (DOCX) [file pone.0169144.s003.docx]

| AMNH | Tad-511 | a Diptera: Ceratopogonidae: *Forcipomyia* sp. ♂ |
| --- | --- | --- |
|  |  | b Diptera: Ceratopogonidae: *Forcipomyia* sp. ♀ |
|  |  | c Diptera: Chironomidae ♀ |
| AMNH | Tad-515 | a Diptera: Ceratopogonidae: *Stilobezzia* sp. ♂ |
|  |  | b Diptera: Sciaroidea |
| AMNH | Tad-616 | a Diptera: Ceratopogonidae: *Forcipomyia* sp. ♀ |
|  |  | b Insect indet. |
| AMNH | Tad-852 | a Diptera: Ceratopogonidae: *Leptoconops* sp. ♂ |
|  |  | b Chironomidae ♀ |
| AMNH | Tad-853 | a *Stilobezzia* sp. ♀ |
|  |  | b Coleoptera |
|  |  | c Coleoptera |
|  |  | d Coleoptera |
|  |  | e Psocoptera |
| AMNH | Tad-858 | a *Meunierohelea orientalis* sp. nov. ♂ |
|  |  | b Coleoptera |
| AMNH | Tad-859 | a *Camptopterohelea* sp. ♀ |
|  |  | b insect indet. |
| AMNH | Tad-862 | a  *Forcipomyia* sp. ♀ |
|  |  | b Psocoptera |
|  |  | c Hymenoptera |
|  |  | d Hymenoptera |
| AMNH | Val-3 * | * This is a large amber flow (with more than 100 syninclusions), which hasn’t been inventoried yet except for the Ceratopogonidae listed in Table 1 and a Limoniidae. |
